# Kinetic Modeling Reveals the Roles of Reactive Oxygen Species Scavenging and DNA Repair Processes in Shaping the Dose-Response Curve of KBrO_3_-Induced DNA Damage

**DOI:** 10.1155/2015/764375

**Published:** 2015-09-10

**Authors:** Maria A. Spassova, David J. Miller, Alexander S. Nikolov

**Affiliations:** ^1^National Center for Environmental Assessment, Office of Research and Development, U.S. Environmental Protection Agency, Washington, DC 20460, USA; ^2^Division of Cancer Biology, National Cancer Institute, National Institutes of Health, Rockville, MD 20892, USA; ^3^Langley High School, McLean, VA 22101, USA

## Abstract

We have developed a kinetic model to investigate how DNA repair processes and scavengers of reactive oxygen species (ROS) can affect the dose-response shape of prooxidant induced DNA damage. We used as an example chemical KBrO_3_ which is activated by glutathione and forms reactive intermediates that directly interact with DNA to form 8-hydroxy-2-deoxyguanosine DNA adducts (8-OH-dG). The single strand breaks (SSB) that can result from failed base excision repair of these adducts were considered as an effect downstream from 8-OH-dG. We previously demonstrated that, in the presence of effective base excision repair, 8-OH-dG can exhibit threshold-like dose-response dependence, while the downstream SSB can still exhibit a linear dose-response. Here we demonstrate that this result holds for a variety of conditions, including low levels of GSH, the presence of additional SSB repair mechanisms, or a scavenger. It has been shown that melatonin, a terminal scavenger, inhibits KBrO_3_-caused oxidative damage. Our modeling revealed that sustained exposure to KBrO_3_ can lead to fast scavenger exhaustion, in which case the dose-response shapes for both endpoints are not substantially affected. The results are important to consider when forming conclusions on a chemical's toxicity dose dependence based on the dose-response of early genotoxic events.

## 1. Introduction

Genotoxicity assays have been widely used to determine qualitatively the carcinogenicity of environmental chemicals in the absence of long term animal studies or epidemiologic data on cancer. Such tests have often led to controversial results [1]. Various types of DNA damage by environmental chemicals have been used as biomarkers for chemical toxicity over the last several decades. DNA damage can be examined using cell or tissue culture and therefore is a much faster and cost efficient assay compared to lengthy animal studies. In recent years, there has been a tendency to extend this approach by using the dose-response of genotoxic events to quantitatively inform cancer risk assessment [2–4]. Johnson et al. [4] advocate the use of a point of departure (PoD) derived from genotoxicity studies* in vivo* and* in vitro* to define a reference dose (RfD) for human risks below which exposure can be considered safe. They summarized methods for defining RfD from genotoxic PoD after the application of the usual scaling and uncertainty factors and using MoA information. The group especially emphasized that sublinear genotoxic dose-responses should be taken into account to define safe levels of carcinogenic chemicals. MacGregor et al. [5, 6] further discuss the need for dose-response analysis of genotoxicity data with specific attention to the assumption of a threshold that defines a safe level of exposure. PoD computed from genotoxicity data is again proposed for use to define risk levels for genotoxic agents [5, 6]. The presence of a threshold in the dose-response of genotoxic events has been argued and experimentally examined in an increasing number of studies [7–9]. A threshold dose-response has also been considered based on a limited amount of genotoxic data for alkylating agents that interact directly with DNA [10]. Special attention has been devoted to DNA repair processes and their protective effect at low doses of exposure which can lead to a threshold [10]. In this commentary, it was recognized that different endpoints that result from exposure to the same chemical can have different dose-response [10]. It is important to point out that most genotoxic data used to demonstrate threshold dose-response are based on acute exposure* in vitro*, not on chronic exposure. The distinction between agents that* directly* interact with DNA and cause DNA adducts and breakage and agents that cause DNA damage* indirectly* has been considered important to define if linear low dose extrapolation should be used [11, 12]. The presence of a threshold in the dose-response dependence has been widely considered for indirect DNA damaging agents [3, 12, 13]. A threshold is expected due to DNA repair and other protective mechanisms. More recently, such thresholds have also been considered and supported by experimental evidence for prooxidants which form DNA adducts [7, 8]. However, the experimental data from these studies are also consistent with low dose linear responses [14]. A distinction between direct and indirect acting genotoxic agents in terms of having a threshold-like dose-response was not confirmed in a systematic study of wide range of genotoxic agents [15]. The analysis of these data did not reveal clear dependence of the dose-response shape on the mode of action [15].

U.S. EPA cancer guidelines [11] recommend the use of dose-response data on precursors (including DNA adducts) to inform the dose-response of chemically induced cancer ([11, Sections 2.3.5.3 and 3.2]). Here we aim to explore to what extent the dose-response of early genotoxic events can be informative for the dose-response determinations of downstream events. Attempts to claim a threshold for carcinogens if some early biomarker, like DNA adducts, presents a threshold are becoming more common [7, 9]. Such a threshold may not be present due to high variability in human population and uncertainty in interspecies extrapolation [16]. However, in addition, there are fundamental pitfalls in trying to translate a threshold of a biomarker into a threshold for a disease. One reason for this is the fact that the experimentally measured biomarker often does not reflect the levels of biomarker initially produced but rather reflects a dynamic equilibrium of the biomarker level. Here, we focus on kinetic modeling to demonstrate that an experimentally defined threshold of an early biomarker may not translate into a threshold for downstream events. We built our model on the example of KBrO_3_, a prooxidant with carcinogenic properties [17–20]. We explore how reliably the dose-response of chemically induced DNA adducts can predict the dose-response of downstream effects. More specifically, we were interested in investigating via kinetic simulation whether the dose-response of DNA adducts that present with threshold-like (sublinear) shape would predict similar dose-responses for other DNA damage processes downstream and could effectively predict the dose-response shape of chemical carcinogenicity. Repairs of DNA adducts, for example, are often argued to define a threshold for the adducts' dose-response and therefore for the cancer's dose-response. Here we demonstrate that since the repair process is not perfect and failed repairs lead to the generation of DNA single strand breaks (SSB), the SSB dose-response can have linear dependence, even when adducts do not. Such a situation, where a DNA adduct's dose-response has a threshold-like behavior, while downstream processes have linear dose-response, is demonstrated for several different scenarios. It is often argued that, in addition to DNA repair processes, detoxification by scavengers is able to counteract effects of environmental chemicals at low doses and determine a threshold for genotoxic events [7, 8, 10]. We demonstrate that, with sustained exposure to toxic chemicals, scavengers of reactive oxygen species and other reactive intermediates can become exhausted even at very low exposure doses. Consequently, the protective effects of scavengers are not preserved with sustained exposure.

## 2. Methods

### 2.1. Kinetic Model of DNA Damage

**Figure 1:**
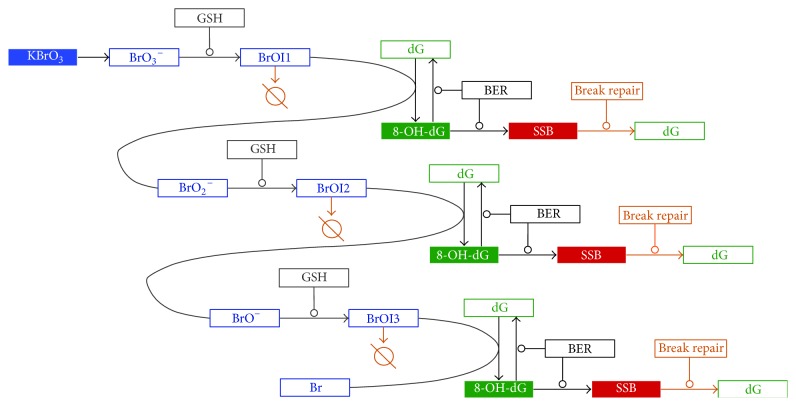
Base model. Our model consists of a series of reactions based on a mechanism proposed by Kawanishi and Murata [17]. Bromate (BrO_3_^−^) reacts with GSH to form a reactive intermediate complex. (BrOI1). This reactive intermediate receives an electron from Guanine, leading to base oxidation and formation of an 8-OH-dG adduct. This damage cycle can repeat twice more with BrO_2_^−^ and BrO^−^, giving each bromate molecule three opportunities to form an adduct. Adduct repair is handled by a base excision repair (BER) mechanism that has a small chance of repair failure resulting in single strand breaks (SSB). A scavenger of the reactive intermediates (*⦰*) and additional SSB repair mechanisms (break repair) were added in specific cases. Round-end arrows indicate enzymatic participation in a reaction.

Here we developed further our kinetic model of KBrO_3_-induced DNA damage [14]. Our aim was to have a realistic model that reflects the experimental evidence in the literature in order to investigate the role of scavengers and DNA repair processes in shaping the dose-response. However, we needed a highly simplified model that can demonstrate the basic features of the dose-response for different types of DNA damage. Therefore, we did not consider special distribution/localization of different compounds or compartmentalization. This model is not intended to be predictive of rates of DNA damage* in vivo* or* in vitro*, as the current understanding of the processes involved is insufficient to support a predictive model. Rather, the model is intended to support further understanding of how different processes involved in this system can interact and how this may influence shapes of dose-response relationships. A number of studies have revealed that KBrO_3_ can cause DNA damage through oxidative stress [8, 17, 23, 24]. It is well documented that at least one of the oxidative DNA damage pathways involves generation of 8-OH-dG adducts. Evidence suggests that KBrO_3_ forms reactive metabolites by interaction with glutathione. Glutathione is considered to undergo redox cycle fast and the redox reactions are not included in our model for simplicity. The reactive metabolites can directly oxidize DNA Guanine residues. In the biochemical model that we developed here after Kawanishi and Murata [17] and used for our simulations, several consecutive oxidation steps are considered (Figure 1). In our model, via interaction with glutathione (GSH), an intermediate product is formed, which can itself form the oxidative lesion 8-OH-dG on DNA. One molecule of bromate can oxidize several Guanine residues in several consecutive steps, where the BrO_3_^−^ ion is reduced to BrO_2_^−^, to BrO^−^, and finally to Br^•^ (Figure 1). The 8-OH-dG lesions can subsequently be repaired by an appropriate base repair mechanism (BER). Alternatively, this repair process can also result in error leading to the production of single strand breaks (SSB), each due to a failed repair attempt, although at a much lower rate than successful repairs (Figure 1) [25]. The 8-OH-dG repair mechanism has been studied using mice knockout model [26, 27]. It has been demonstrated that the knockout Ogg1−/− mice have elevated mutation rates in proliferating liver cells due to a higher presence of 8-OH-dG after exposure to KBrO_3_ [26], suggesting involvement of OGG1 in the repair process. Furthermore, there is some evidence that KBrO_3_ can directly cause SSB [28], but, for simplicity, we did not include that pathway in our model. The schematics in Figure 1 reflect the biochemical reactions that are included in our computational model in the form of differential equations. The reactive intermediates labeled here BrOI1, BrOI2, and BrOI3 can oxidize Guanine residues to form 8-OH-dG. In some versions of the models we have also included an additional SSB repair mechanism in order to investigate how this step can affect the dose-response dependence of 8-OH-dG and SSB levels. The model elements Guanine, GSH, and BER were given appropriate initial values and KBrO_3_ was dosed at time zero. The reactions were modeled using simple mass action kinetics, with all simulations carried out using MATLAB SimBiology software. The list of the chemical reactions included in the model and modeled as a system of differential equations is as follows:

(1) KBrO3⟶BrO3−GSH+BrO3−⟷GSH·BrO3−GSH·BrO3−⟶GSH+BrOI1BrOI1+S⟶BrOI1Sscavenger  variant  onlyBrOI1+Guanine⟷BrOI1·GuanineBrOI1·Guanine⟶BrO2−+8-OH-dG8-OH-dG+BER⟷8-OH-dG·BER8-OH-dG·BER⟶BER+Guanine8-OH-dG·BER⟶BER+SSBSSB+BR⟷SSB·BRBreak  Repair  variant  onlySSB·BR⟶BR+GuanineBreak  Repair  variant  onlyGSH+BrO2−⟷GSH·BrO2−GSH·BrO2−⟶GSH+BrOI2BrOI2+S⟶BrOI2Sscavenger  variant  onlyBrOI2+Guanine⟷BrOI2·GuanineBrOI2·Guanine⟶BrO−+8-OH-dGGSH+BrO−⟷GSH·BrO−GSH·BrO−⟶GSH+BrOI3BrOI3+S⟶BrOI3Sscavenger  variant  onlyBrOI3+Guanine⟷BrOI3·GuanineBrOI3·Guanine⟶Br+8-OH-dG

where the dot notation signifies a bound complex of two participants, S is scavenger, BROI1S, BROI2S, and BROI3S are inactive compounds that cannot oxidize DNA and are removed from the system, labeled as (*⦰*) in Figure 1, BER is base excision repair mechanism, and BR is break repair mechanism. The MATLAB script is available upon request.

### 2.2. Statistical Analysis

Dose-response analysis was carried out using EPA's benchmark dose software (BMDS). The results were plotted using Origin software (Figure 6). We used a likelihood approach to evaluate the dose-response models' goodness-of-fit.

## 3. Results and Discussion

Our objectives were motivated by the tendency in the literature to assume that a threshold in early upstream events would define a threshold in downstream events. More specifically, it is often considered that a threshold in a genotoxic event suggests a threshold for a related adverse effect like cancer. Here we investigated how the dose-response of a downstream endpoint is defined by an upstream endpoint with threshold-like dose-response shape. As an early upstream event, we modeled the generation of DNA adduct 8-hydroxy-2-deoxyguanosine (8-OH-dG) by the prooxidant KBrO_3_. DNA adducts formed by oxidation are often used as important biomarkers of oxidative stress in risk assessment, and it is of interest to investigate how the dose-response of this biomarker defines the dose-response of downstream events. The generation of single strand breaks (SSB) was modeled here as a downstream event due to failed DNA repair of the adducts (Figure 1). Various conditions are thought to provide a protective effect at low doses of exposure to ROS, including the presence of antioxidants, DNA repair processes, or depletion of metabolizing agents that activate prooxidants [29]. We investigated how different scenarios may affect the dose-response dependence of early DNA damage events and if a threshold-like dose-response is to be expected for different endpoints. Using kinetic modeling, we have demonstrated that under a broad range of conditions the downstream SSB dose-response can remain largely linear, even when upstream 8-OH-dG shows a concave up, threshold-like dose-response behavior. More specifically, we investigated how the dose-response shape would be affected by base excision repair and single strand break repair (BER/SSBR) mechanisms, reduced glutathione (GSH), or a terminal scavenger of the bromate reactive intermediates.

### 3.1. Basic Model

**Figure 2:**
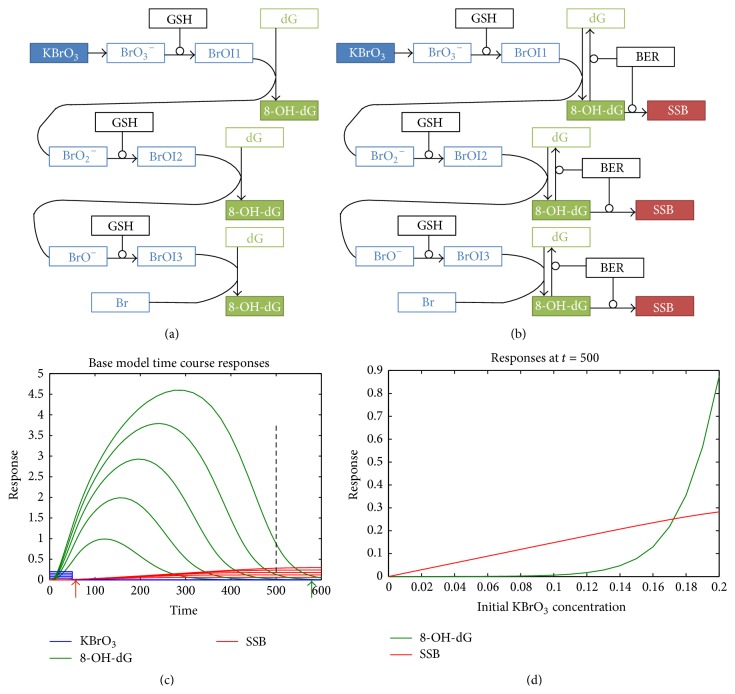
Base model simulation results. (a) Model used for simulation of 8-OH-dG formation in absence of any DNA repair mechanisms. (b) Base Model used for simulations of DNA adducts and SSB formation in the presence of a BER repair mechanism. (c) Time course of KBrO_3_ exposure, 8-OH-dG, and SSB levels determined by simulations using the model in (b). A brief period of KBrO_3_ exposure (blue) in the presence of GSH, followed by quick KBrO_3_ removal. A base excision repair (BER) mechanism is active (b). The time course of 8-OH-dG adducts (green) shows a fast initial increase of adducts and consequent decrease as the BER repairs the adducts. However, infrequent repair failure results in persistent single strand breaks (SSB) that gradually accumulate over the course of the simulation (red). (d) A dose-response plot sectioned from a time point marked in (c) (dashed line) shows that the SSB response can be linear despite the nonlinear, threshold-like appearance of the 8-OH-dG adducts.

**Figure 3:**
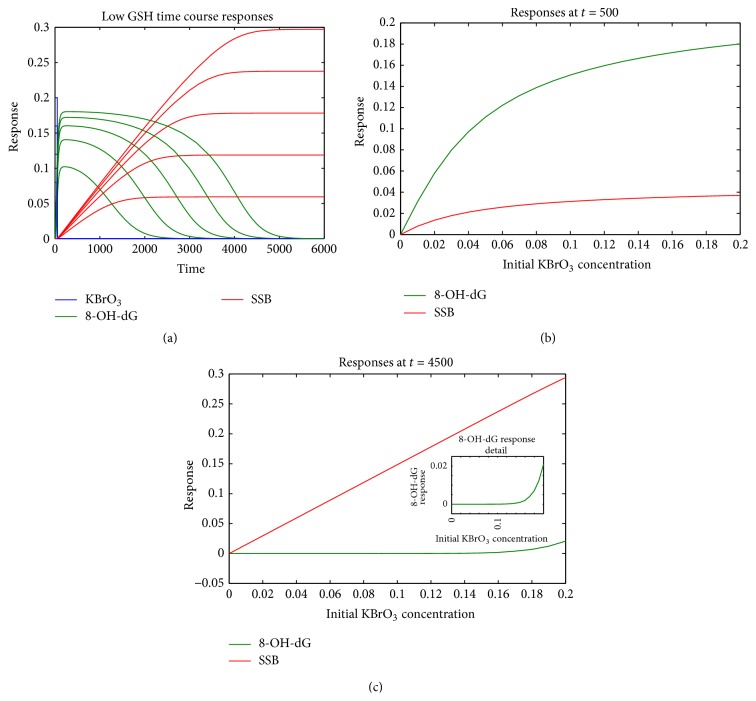
Effect of GSH levels on KBrO_3_-induced oxidative DNA damage. GSH levels were drastically reduced in this simulation to reveal how different concentrations of GSH would affect the dose-response dependence of 8-OH-dG and SSB. (a) Time dependence of 8-OH-dG (green) and SSB (red) levels after exposure to various concentrations of KBrO_3_ (blue). (b) Dose-response dependence of 8-OH-dG (green) and SSB (red) short time after exposure (*t* = 500). (c) Dose-response dependence of 8-OH-dG (green) and SSB (red) at a time point shortly before steady state, reached much later after the exposure, reveals dose-response shapes of 8-OH-dG (inset) and SSB similar to the base model.

**Figure 4:**
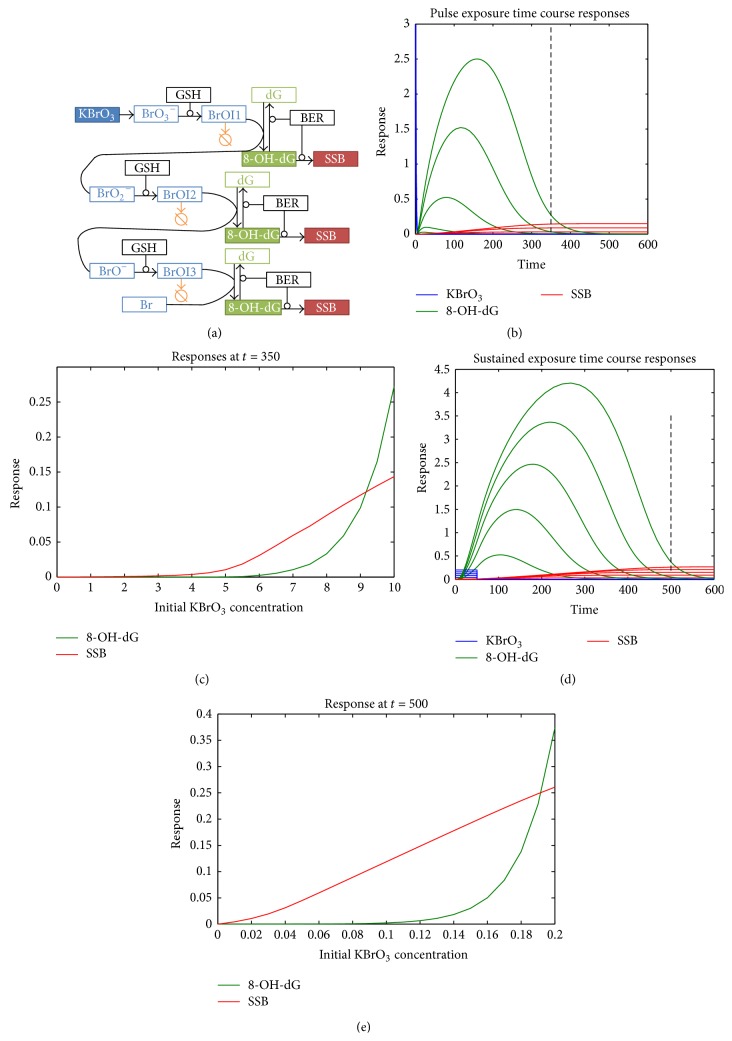
Scavenging of KBrO_3_/GSH complex reactive intermediates. (a) A scavenger was included in the model as shown. (b) and (c) Pulse exposure to KBrO_3_ in the presence of a scavenger. (b) Time dependence of 8-OH-dG (green) and SSB (red) levels after pulse exposure to KBrO_3_ (blue). (c) Dose-response dependence of 8-OH-dG (green) and SSB (red) at a time point shown in (a) with vertical dashed line (*t* = 350). Both 8-OH-dG and SSB have highly sublinear threshold-like dependence. (d) and (e). Sustained exposure to KBrO_3_ in the presence of a scavenger. (d) Time dependence of 8-OH-dG (green) and SSB (red) levels after exposure to various concentrations of KBrO_3_ (blue). (e) Dose-response of 8-OH-dG and SSB at *t* = 500 is similar to that in the base model due to exhaustion of the scavenger.

We initially modeled the dose-response of 8-OH-dG in the absence of BER and SSB repair mechanisms (Figure 2(a)). The simulation revealed a linear increase of 8-OH-dG with exposure to increasing KBrO_3_ levels, as expected (data not shown). In the next step, a BER mechanism was added to the model and SSB were generated when repair was not completed (Figure 2(b)). For this model, a brief exposure to different levels of KBrO_3_ was applied. The time course of KBrO_3_ exposure, 8-OH-dG formation, and SSB formation is plotted on Figure 2(c). Five levels of KBrO_3_ concentration within the range we used in the simulation are plotted (blue) with the corresponding time course of 8-OH-dG levels (green) and SSB levels (red). A similar format is used in the consecutive figures showing time course of KBrO_3_ exposure, 8-OH-dG levels, and SSB levels (Figures 3(a), 4(b), and 4(d)). We used only relative units of time as we did not attempt to predict the actual time course but we were rather interested in the resulting shape of the dose-responses of 8-OH-dG and SSB for different scenarios. Initially, the 8-OH-dG lesions increased steeply as the repair lags behind. However, shortly after the end of the exposure, the 8-OH-dG lesion levels steeply decreased due to successful and failed repair. The SSB continuously accumulated with time as the rates of failed repair were kept constant (Figure 2(c)). It is important to note that if experimental measurements are made at a time shortly after the exposure (red arrow), the SSB would not be detected for the entire KBrO_3_ concentration range. On the other hand, measurements made a long time after the exposure would not be able to detect 8-OH-dG lesions (green arrow). These results can explain to some extent discrepancies between different experimental studies. The dose-response dependence of 8-OH-dG and SSB was plotted at Figure 2(d) at an intermediate time after exposure (*t* = 500; the dotted line in Figure 2(c)). The simulation of the 8-OH-dG dose-response revealed highly sublinear, threshold-like behavior. At low KBrO_3_ doses repair of 8-OH-dG lesions completes, creating a dose threshold for KBrO_3_ effects. It is also important to mention that in this scenario 8-OH-dG would not be a suitable measure of exposure and should not be used as an exposure biomarker, as it may not be detected if measured a long time after exposure, even after exposure to high concentrations of KBrO_3_. However, SSB generated downstream from the 8-OH-dG, accumulated over time, and the dose-response dependence for SSB is strictly linear (Figure 2(d), red) suggesting that consecutive downstream points may have preserved linear dependence.

### 3.2. Depletion of GSH

We modified the model to reflect various possible physiological situations. First, the role of GSH as a catalyzing agent for the DNA oxidation by bromate was examined. In our model, the GSH concentration was drastically reduced so that GSH binding to KBrO_3_ became rate-limiting. Accordingly, with a similar protocol of exposure to KBrO_3_, the generation of 8-OH-dG lasted for much longer time after the exposure, reached much lower maximal levels, and overlapped more significantly with repair. These effects are clearly identified when comparing the time course and the response amplitude of 8-OH-dG plotted on Figures 2(c) and 3(a) (note different scales). The steady state levels of SSB were similar but were reached at much later time point when GSH was depleted. The dose-response of both 8-OH-dG and SSB is plotted on Figures 3(b) and 3(c) at two different time points (*t* = 500 and *t* = 4500). Early after exposure, the dose-response of 8-OH-dG had a concave down shape (Figure 3(b)) rather than the concave up shape in our base model. This is due to lower increase of 8-OH-dG generation rates with bromate concentration at higher concentrations of bromate and is not related to the repair. Therefore, early on after the exposure, the 8-OH-dG dose-response flattened out at higher doses of KBrO_3_ (Figure 3(b)). The fact that generation of 8-OH-dG adducts lasted for much longer time is manifested by the high levels of 8-OH-dG adducts even at *t* = 3000 (Figure 3(a)), long after all the adducts were repaired in our base model (Figure 2(c)). However, after sufficient amount of time, when all the bromate was depleted, the generation of new 8-OH-dG adducts ceased and 8-OH-dG repair was close to completion (*t* = 4500); 8-OH-dG had similar threshold-like dose-response shape as in our base model (Figure 3(c)). As the SSB accumulated due to failed repair, a linear dose-response dependence of SSB is also once again observed (Figure 3(c), red). Overall, the shape of the dose-response of both 8-OH-dG and SSB at a time point approaching the steady state with this model simulation was similar to our base model. These results once again demonstrate that the experimental measurements can have very different outcomes depending on combination of factors, where crucial factors are the time after exposure, and whether the exposure is sustained. If experimental measurements are taken at steady state level, 8-OH-dG may not be detected at all. Nevertheless, SSB levels at low levels of exposure would increase linearly with dose.

### 3.3. Presence of Scavenger

Furthermore, we investigated how an effective antioxidant (scavenger) of the bromate reactive intermediate can modify the dose-response dependence of 8-OH-dG and SSB. There is experimental evidence that a terminal (or suicidal) scavenger, such as melatonin, can have a protective role in bromate-induced oxidative stress [30]. A terminal scavenger cannot undergo repeated reduction and oxidation because it forms a stable product when oxidized. While other antioxidants can themselves become prooxidants and cause oxidative damage, terminal scavengers are depleted once oxidized, and they cannot further serve as antioxidants. For simplicity, we included a terminal ROS scavenger rather than an antioxidant that can become prooxidant and has to be accounted as such. The effect of a terminal scavenger added to the system is shown in Figure 4. In the case of brief exposure to KBrO_3_ (Figures 4(b)-4(c)), the scavenger effectively prevented generation of 8-OH-dG at low KBrO_3_ concentrations. Of the five levels of 8-OH-dG and SSB corresponding to increasing KBrO_3_ concentrations plotted on Figure 4(b), the lowest levels of 8-OH-dG and SSB were very minimal and invisible at this scale. The next KBrO_3_ concentration level also only generated a low amount of DNA damage (Figure 4(b)). Therefore, there was no SSB accumulation due to failed 8-OH-dG repair at low KBrO_3_ concentrations. Accordingly, our simulation predicted clear threshold-like dose-response curves for both 8-OH-dG and SSB, where there is no DNA damage with exposure to low KBrO_3_ concentrations (Figure 4(c)). It is important to point out, however, that the visually defined dose threshold of the SSB dose-response (Figure 4(c), red) is lower than the dose threshold of 8-OH-dG. This can be recognized by the shift of the SSB dose-response steep-increase phase to the left (to the lower concentrations of KBrO_3_) relative to the 8-OH-dG dose-response steep phase. A visually defined threshold is at about 3.5 [KBrO_3_] for SSB versus 5.5 [KBrO_3_] for 8-OH-dG. This result is important to consider when a quantitatively defined threshold of early genotoxic event is considered to be used for defining safe levels of adverse endpoints such as cancer. Furthermore, the protective effects of the scavenger are completely erased with prolonged exposure to KBrO_3_ (Figures 4(d)-4(e)). Prolonged exposure to KBrO_3_ leads to scavenger exhaustion. Therefore, with prolonged exposure, even very low concentrations of KBrO_3_ were able to cause DNA damage by generating 8-OH-dG. As a consequence, SSB were accumulated even at low KBrO_3_ concentrations. Due to both successful and failed repair of 8-OH-dG, the dose-response of 8-OH-dG had a threshold-like dependence (Figure 4(e)), while the SSB accumulated with failed repairs and showed close to linear dependence, with clear low dose linear increase, even in the presence of scavenger. Overall, even when an effective scavenger is present, with prolonged exposure to KBrO_3_, the dose-response curves of 8-OH-dG and SSB are similar to those in our base model at a time point close to the steady state. It is important to note that, in all cases considered so far, at steady state (at the end of the time period shown) when the 8-OH-dG repair is completed, the adducts cannot be detected even at high levels of exposure, whereas the SSB still have linear dependence.

### 3.4. Single Strand Break Repair

**Figure 5:**
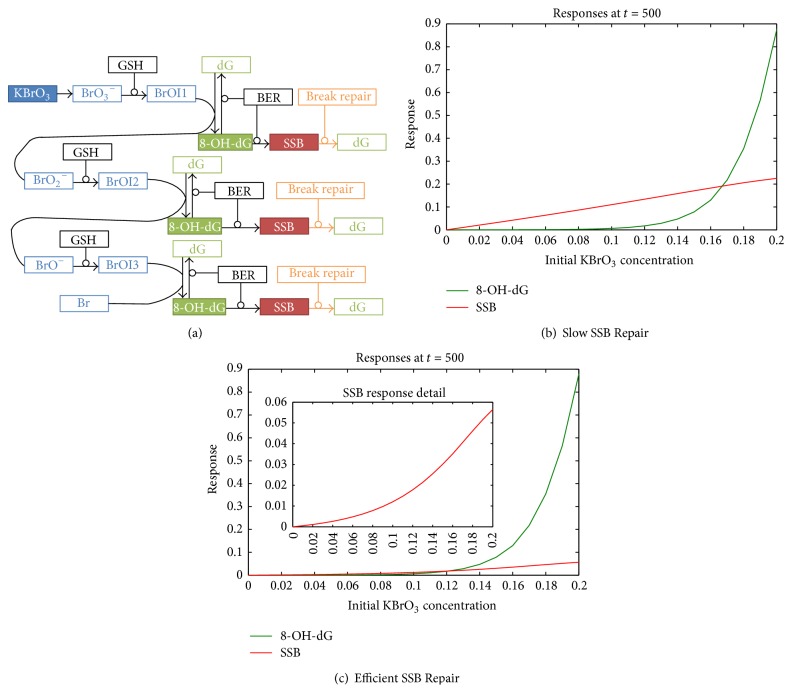
Simulations with an additional SSB repair (SSBR) mechanism. (a) An additional SSB repair mechanism was included in the model. (b) SSB repair rate constant was set to be slower than BER. In this case the dose-response of 8-OH-dG was highly sublinear, while the SSB dose-response had linear behavior as in our base model. (c) SSB repair rate constant was set to be similar to the BER rate constant. Dose-response dependence of 8-OH-dG and that of SSB (inset) both had some level of sublinearity.

Finally, we considered the possibility that SSB formed by a failed BER attempt is later detected and repaired by other SSB repair pathways. SSB repair (SSBR) most often includes several steps [25]: (1) SSB detection; (2) DNA end processing; (3) DNA gap filling; and (4) DNA ligation. There is a possibility of failure at any of these steps. Failure at different stages of these processes can have different outcomes, including single point mutations and deletions. For simplicity, we did not include all of these possible failure processes in our model and included only efficient SSB repair. First, we considered the case when the rate of SSB repair is low compared to the rate of SSB generation by failed BER. In this case, with sustained exposure to bromate, the 8-OH-dG adducts were efficiently repaired at low concentrations of bromate exposure and resulted in highly sublinear, threshold-like dose-response dependence as in our base model. However, when the rate of SSBR is slower, the SSB accumulate and the dose-response dependence of SSB remains linear (Figure 5(b)), again similar to our base model. Under another scenario, we considered similar rates of SSB formation and repair. In this case both dose-response curves had some sublinearity (Figure 5(c)). Overall, the shape of the SSB dose-response was defined by the relative rate of the SSBR compared to the BER. Highly efficient SSB repair can increase the sublinearity of the SSB dose-response to a threshold-like behavior.

Failed SSBR can lead to mutations as downstream events [31]. As discussed above, there are a number of different pathways and mutation types that can occur. Mutations are downstream of SSB, similar to the SSB being downstream of the 8-OH-dG adducts. It is conceivable that even when SSB are completely repaired at low doses and show threshold-like dose-response the cumulative mutation rates can still show linear dose-response dependence. Since the mutations are irreversible and cannot be repaired, they persist and can lead to other adverse effects at low doses.

**Figure 6:**
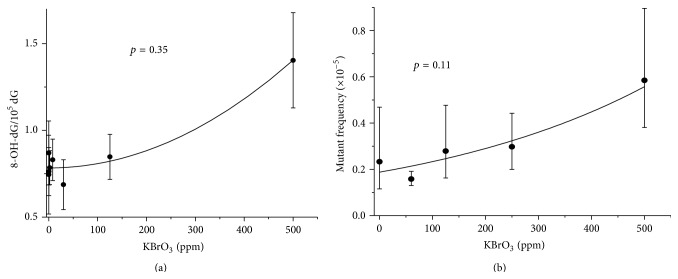
Dose-response of genotoxic events in the kidney of rats exposed to KBrO_3_. Data on kidney were selected as the kidney is the target organ of KBrO_3_ carcinogenicity. (a) Data on 8-OH-dG levels in DNA extracted from the kidneys of rats exposed to KBrO_3_ [9] are plotted versus KBrO_3_ concentration. Data are fitted with a quadratic model with a zeroed linear term. (b) Deletion mutations in the kidneys of gpt delta rats exposed to KBrO_3_. Dose-response data from Umemura et al. [21] are plotted. An exponential, low dose linear, model ([22, model 2]) is fit to the data for Spi^−^ mutation frequency as a measure of deletion mutations. A lognormal distribution of the data at each concentration was assumed and a log-scale constant variance model was used.

We previously investigated if the presence of a threshold in dose-response dependence can be confirmed statistically by fitting various models to genotoxic data. We and others concluded that most dose-response data that show a high level of sublinearity are consistent with both threshold models and low dose linear models [14, 32]. Therefore, we did not attempt to fit our kinetic model to experimental data to show that one set of adducts data has a threshold, while a downstream event does not. Rather we provide an example, where data are qualitatively consistent with our kinetic modeling predictions. Here we analyzed data from Yamaguchi et al. [9] and Umemura et al. [21] on 8-OH-dG adducts and Spi^−^ mutations in the kidney of rats exposed to KBrO_3_ through their drinking water (Figure 6). The 8-OH-dG adducts data were consistent with a quadratic model with goodness-of-fit, *p* = 0.35 (Figure 6(a)). The model is highly sublinear and has zero slope at low doses (with a zeroed linear term) and therefore the fit is suggestive of a threshold. The Spi^−^ mutants in gpt delta rats [21] are considered an event downstream the 8-OH-dG adducts after exposure to KBrO_3_ [14]. The data on Spi^−^ mutants in rat kidney were consistent with low dose linear model, where the frequency at low doses increased linearly with dose (Figure 6(b)). Visually, this difference is clear when looking at the mean response to 125 ppm KBrO_3_ exposure, for example. The mean value at 125 ppm for the adducts is not higher than the mean response value of the lower doses, while the mutant frequency at 125 ppm is higher than the response values of the lower doses.

As we discussed earlier, a clear mathematical proof of a threshold of a dose-response dataset is difficult for most datasets due to data variability. Generally, a threshold for a dataset can be determined by a fit with a threshold model [33]. “Threshold” in the context of our simulation refers to a dose (or dose region) at which a transition from a flat line (with no increase with dose) to a steep phase occurs. A “threshold-like dose-response” refers to a highly sublinear dose-response with transition from flat phase to steep phase.

Overall, we demonstrate here that the results from our base model are valid for wide range of physiological conditions and consistent with data in the literature. The results from our kinetic simulation would warn against using a threshold determined by the dose-response of an early genotoxic event as a safe level, because downstream events may have lower dose threshold or no threshold at all.

Efforts to use early biomarkers' dose-response to determine levels of chemical exposure that do not have adverse health effects are widely reflected in the literature. Genotoxic assays have been widely used for the qualitative determination of carcinogenicity of environmental chemicals. Efforts to expand the use of such tests in a quantitative manner to determine levels of exposure to environmental chemicals and pharmaceutical agents without adverse health effects are rising. The report of the Genetic Toxicology Technical Committee (GTTC) Quantitative Analysis Workgroup (QAW) [4] considered methods for quantitative use of genotoxicity data. The group reviewed methods for direct use of a point of departure (PoD) defined from genotoxicity data to calculate a reference dose (RfD) of human exposure below which adverse health effects are unlikely. The report proposed calculation of RfD from the PoD after the application of the usual scaling and uncertainty factors. In light of our kinetic modeling, such direct extrapolation has to be performed carefully and may not be appropriate in some cases, as early genotoxic events may not be detected at low doses, while downstream effects may be present. As we demonstrated, with short periods of chemical exposure, a downstream effect may have dose-response which is still sublinear but with a threshold-like transition at much lower doses than an upstream biomarker (Figure 4(c)). Our results also show that, with prolonged exposure, even under various protective conditions, early genotoxic event may have threshold-like dose-response that will determine high PoD, while downstream event may have close to linear dose-response with much lower PoD (Figure 4(e)).

Our simulation can help explain experimental results from different genotoxicity assays.* In vitro* genotoxicity tests, when used alone, have up to 41% false negative results [1]. This situation is entirely different when several genotoxicity tests are combined and a positive result is reported when being positive in at least one assay. For some assay combinations (e.g., Ames + micronucleus test (MN)) the sensitivity is elevated to 94%, which corresponds to false negative rate of 6% [1]. These studies demonstrate high discrepancy among different genotoxicity tests* in vitro* and an inability of these tests alone to predict accurately the carcinogenicity potential of chemicals. An* in vitro* assay that reflects more complex cell responses, the Syrian Hamster Embryo (SHE) assay, has much higher concordance with rodent two-year bioassays (89%), even though it cannot differentiate between rodent and human carcinogens [34, 35]. In general, many factors can affect the experimental outcome. For example, it has been previously shown that actively transcribed genes are repaired faster than other DNA regions [36]. Therefore, it is important to consider what regions of DNA have been evaluated for DNA damage. Our results highlight some other possible reasons for the high discrepancy between genotoxic assays. Our results demonstrate that a genotoxic response, like DNA adduct formation, may not be present at low doses, while downstream genotoxic events, like SSB formation, can be present at the same low doses. Our study may help explain controversial results from genotoxicity data of chemicals. The simulation we performed further demonstrates that false negative results can arise due to (1) short periods of exposure in the presence of scavenger as the simulations with this scenario showed that the responses of both SSB and 8-OH-dG are suppressed in the lower half of the dose range (Figure 4(c)), (2) the fact that DNA SSB/DSB levels are measured shortly after exposure when they have not yet accumulated due to failed repairs (Figure 2(c), red arrow), and (3) the fact that 8-OH-dG DNA adducts' levels are measured a long time after a brief exposure when repair has already taken place (Figure 2(c), green arrow).

## 4. Conclusion

Overall, we believe our mathematical examination of the dose-response of early genotoxic biomarkers is critical to consider when a biomarker dose-response is used to define dose dependence of chemical exposure adverse effects. As we demonstrated with our computational approach, downstream events may have lower thresholds or no threshold at all. We anticipate that our conclusions are valid in more general terms for other upstream biomarkers but recognize that further studies are needed to examine this hypothesis. The simulation presented here reveals how computational methods that describe biochemical pathways can be used to inform risk assessments.

We recommend careful consideration of dose-response data at several precursor event levels in conjunction with data on cancer incidence (if available) to properly identify carcinogenicity of environmental chemicals and define their dose dependence. Our results demonstrate that using the dose-response of a single biomarker/precursor event to define dose dependence of related adverse effects due to chemical exposure is not necessarily appropriate. A more careful and complex experimental paradigm is needed to characterize the multistage process of chemical carcinogenicity and other adverse effects in a quantitative manner.

## Abbreviations

KBrO_3_: Potassium bromate
ROS: Reactive oxygen species
MN: Micronucleated cells assay
8-OH-dG: 8-Hydroxy-2-deoxyguanosine
GSH: Glutathione
BrOI: Reactive oxygen intermediates formed by KBrO_3_ interaction with GSH
BMDS: EPA's benchmark dose software
OGG1R: Ogg1 initiated base excision repair mechanism
SSB: Single strand break
SSBR: Single strand break repair
DSB: Double strand break
PoD: Point of departure
RfD: Reference dose
SHE: Syrian Hamster Embryo
GTTC: Genetic Toxicology Technical Committee
QAW: Quantitative Analysis Workgroup.
